# “Heartbreak and Frustration’’ experiences of healthcare providers caring for pregnant women with sickle cell disease in Uganda

**DOI:** 10.1371/journal.pone.0355018

**Published:** 2026-08-07

**Authors:** Jackline Akello, Annettee Nakimuli, Grace Ndeezi, Musa Sekikubo, Ian Munabi, Ruth Namazzi, Deogratias Munube, David Mukunya, Francis Pebalo, Kenneth Mugabe, Savio Mwaka, Sarah Kiguli, Joseph Rujumba

**Affiliations:** 1 Department of Obstetrics and Gynaecology, School of Medicine, College of Health Sciences, Makerere University, Kampala, Uganda; 2 Department of Pediatrics and Child Health, School of Medicine, College of Health Sciences, Makerere University, Kampala, Uganda; 3 Department of Anatomy, School of Medicine, College of Health Sciences, Makerere University, Kampala, Uganda; 4 Busitema University, Mbale, Uganda; 5 Department of Obstetrics and Gynecology, School of Medicine, Gulu University, Gulu, Uganda; 6 Department of Obstetrics and Gynecology, Mbale Regional Referral Hospital, Mbale, Uganda; 7 Infectious Diseases Research Collaboration, Kampala, Uganda; King Fahd Military Medical Complex, SAUDI ARABIA

## Abstract

**Introduction:**

Sickle Cell Disease (SCD) is a hereditary hemoglobinopathy that poses significant risks during pregnancy, leading to increased maternal complications such as Vaso-occlusive crises, acute chest syndrome, preeclampsia, preterm labor, and perinatal complications such as preterm births, low birthweights and mortality. The complexities surrounding the management of SCD extend beyond the clinical symptoms and require a comprehensive understanding of the social, emotional, and systemic factors that influence patient outcomes**.** While existing research has largely focused on the epidemiology and clinical outcomes of SCD in pregnancy, little is known about the perspectives of frontline healthcare providers in resource-limited settings.

**Objective:**

To explore the experiences of healthcare providers in managing pregnant women with sickle cell disease.

**Methods:**

A qualitative approach with phenomenological research design was employed, utilizing in-depth interviews and Focus Group Discussions (FGDs) to gather insights from healthcare providers at a regional and national referral hospital. Participants included obstetricians, midwives, and general practitioners working in maternity departments. Inductive thematic analysis was conducted to identify key themes and patterns.

**Results:**

The study identified several themes based on various health system building blocks and the different levels of the Behavioural Ecological Framework. Individual-level themes included knowledge gaps and awareness, fear and anxiety related to managing a pregnant woman with SCD, and emotional and psychological distress. Community-level influences consisted of religious beliefs, cultural beliefs, as well as myths and misconceptions. At the health systems level, key themes encompassed diagnostic challenges, shortages of critical supplies, lack of management guidelines, and the absence of multidisciplinary and specialized services.

**Conclusion:**

A multidisciplinary approach that integrates clinical expertise, patient education, and healthcare system reforms is essential for improving maternal and neonatal health in pregnant women with SCD.

## Introduction

Sickle Cell Disease (SCD) is a hereditary blood disorder characterised by the production of abnormal haemoglobin, leading to the deformation of red blood cells into a sickle shape. This condition presents significant health challenges, especially during pregnancy, when physiological changes can exacerbate complications associated with SCD. Globally, SCD affects approximately 5% of the population, with the highest prevalence found in sub-Saharan Africa, India, and the Middle East [1]. The World Health Organisation (WHO) has recognised the need for improved healthcare strategies for SCD management, particularly in regions where the disease is endemic [2]. The Lancet Haematology Commission has made specific recommendations aimed at enhancing care for individuals with SCD, emphasising the importance of integrating disease management into primary healthcare systems [3]. These recommendations underscore the necessity of a multidisciplinary approach that includes healthcare providers, social support systems, and patient education to mitigate the risks associated with SCD, particularly during pregnancy.

Pregnancy in women with SCD presents significant risks, including heightened rates of maternal morbidity and mortality, preterm birth, and fetal complications [4]. The physiological changes that occur during pregnancy, such as increased blood volume and alterations in blood viscosity, can trigger Vaso-occlusive crises and other complications related to SCD. Therefore, it is essential to understand the unique challenges faced by healthcare providers in managing SCD during pregnancy and the implications of these challenges on maternal and fetal outcomes.

In Uganda, SCD is a significant public health issue, affecting an estimated 1.5% of the population, with a higher prevalence observed in the Eastern and Northern regions of the country [5]. The healthcare delivery in Uganda is constrained by inadequate resources, limited access to specialised care, and a lack of comprehensive SCD management protocols. These challenges are compounded for pregnant women with SCD, who require specialised care to navigate the complexities of their condition during pregnancy.

The role of healthcare providers in managing SCD during pregnancy is crucial. They require adequate knowledge, resources, and effective communication skills to provide comprehensive care that addresses both medical and psychosocial needs, ensures appropriate prenatal care, education and support, and enables timely responses to pregnancy-related complications to prevent adverse outcomes.

While existing research has largely focused on the epidemiology and clinical outcomes of SCD in pregnancy, little is known about the perspectives of frontline healthcare providers in resource-limited settings. Healthcare provider experiences are critical for understanding how systemic gaps translate into day-to-day challenges and for identifying opportunities to strengthen care. This study, therefore, explored healthcare providers’ experiences in managing pregnancy complicated by SCD at Kawempe National and Mbale Regional Referral Hospitals in Uganda

## Materials and methods

### Study design, setting and study population

This qualitative study employed a phenomenological design using in-depth interviews (IDIs) and focus group discussions (FGDs) with health workers involved in the care of pregnant women with Sickle cell disease at Kawempe National Referral Hospital (KNRH) and Mbale Regional Referral Hospital (MRRH) from 8^th^ of January to March 30th 2025. KNRH is a tertiary health facility in Kampala, the Capital city of Uganda. KNRH provides referral care for women in its catchment area and those referred from across Uganda in need of specialized care, including those with SCD. The KNRH annual report for 2023 showed that an estimated number of about 140 pregnant women with SCD sought different services in the hospital including ANC, labor and delivery.

MRRH, a tertiary facility located 245 km East of Kampala, serves 16 districts with a catchment population of more than 4.5 million people and receives an estimate of two to five new cases of SCD weekly in the SCD clinic which is for both pregnant women and the general population.

Participants included intern doctors, midwives, residents in Obstetrics and Gynaecology, medical officers, anesthesiologists, and obstetricians. Purposive sampling was used to ensure representation across cadres and units that provide care to women with SCD, including antenatal clinics (ANC), intensive care units (ICU), obstetric high dependency unit (HDU), labor wards and theatres. The sample size was determined by data saturation.

### Eligibility criteria

Health professionals directly involved in the care of pregnant women with SCD and with at least six months of relevant experience were included. All participants provided written informed consent.

### Ethical standards and approvals

The study obtained approvals from the Institutional Review Board (IRB) of Makerere University, School of Medicine Ethical Review Board, approval number Mak-SOMREC-2023–805, as well as approvals from the Uganda National Council for Science and Technology (UNCST), number HS5315ES and administrative clearance from both Kawempe National Referral Hospital and Mbale Regional Referral Hospital.

The study complied with ethical standards as outlined in the Belmont Report and the Declaration of Helsinki. Each participant also provided informed written consent and signed a consent form before participating in the study. Minors below the age of 18 years were not included in the study. Each participant was given a token of appreciation for time compensation worth twenty thousand (20,000) Ugandan Shillings after the interviews and/or FGDs as approved by the IRB. To ensure confidentiality of the participants, during the recording, personal identifiers were not included and after the recordings were stored by each researcher independently with limited access. The recordings were then shared with the Principal Investigator (PI) for transcription. The PI ensured that all transcripts had no identifiers and were stored in the research computer which is protected by password with restricted access. No participant declined to participate in the study.

### Study procedure and sampling

The study was conducted in a phased approach with a total number of 34 participants from 14 IDIs and four FGDs. The FGDs were composed of six members per group, at KNRH and four members per group at MRRH. The difference in the number of participants in both the IDIs and FGDs were due to the difference in staffing norms between the two hospitals in terms of numbers and cadres. While KNRH has an anaesthesiologist whose experience and view are critical for these vulnerable population, MRRH lacks one. On the other hand, whereas MRRH has Medical Officers who are also primary care givers, KNRH structure does not include this category. The purposive sampling was therefore to ensure that all cadres were captured in the interviews and then some involved in the FGDs. Both facilities lack haematologists who are critical for the management of patients with SCD. IDIs were conducted first to capture individual experiences, followed by FGDs to explore shared or divergent perspectives. Individual interviews lasted 45–60 minutes, took place in private in outpatient rooms. The FGDs took place in the staff boardroom and lasted 1.5 to 2 hours. The interviews were conducted by two research assistants trained in qualitative data collection. Sessions were audio-recorded, transcribed and de-identified to remove confidential information. A reflective journal and field notes were also used by the research team, adding rigour to the qualitative inquiry. Each investigator recorded her reactions, assumptions, expectations, and biases about the research process. The field notes also provided additional data for the analysis. To minimize power imbalances and social desirability bias, the PI did not conduct interviews with healthcare providers, as she was known to them in a clinical capacity. Instead, interviews were conducted by an independent qualitative interviewer and supported by research coordinators trained in qualitative research methods. Reflexivity was addressed through repeated reading of transcripts, team-based discussions of emerging findings, and engagement with other qualitative researchers to critically examine interpretations and enhance analytic rigor.

### Data management and analysis

Data collection and analysis occurred concurrently, with emerging themes informing subsequent discussions. The data was collected in a phased approach with IDIs where we had IDIs being done first with ensure each cadre was represented and achieved saturation after 14 IDIs. Following that, the team then embarked on FGDs to obtain any group ideas or divergent views that may not have emerged from the IDIs and saturation was achieved after four FGDs. The transcripts were reviewed by the PI, study coordinator and an independent study analyst. Coding was done manually by a single coder with review by the PI and a qualitative data specialist. The coding was inductive as there were no preset themes. The codes were then aligned into themes and sub-themes guided by the behavioural ecological framework (BEF) for examining healthcare access and navigation [6], complemented by the World Health Organisation’s health system building blocks. The BEF provides a comprehensive lens to examine healthcare access, particularly in the context of managing SCD. This multi-level framework allows for the exploration of individual, community, policy, and health system factors that influence healthcare access and outcomes. Themes and sub-themes that emerged during data collection were refined following multiple readings of the interview transcripts, enabling the identification and grouping of data into the different levels of the framework.

## Results

### Participant demographics

A total of 34 healthcare providers participated in this study, 12 from Mbale Regional Referral Hospital and 22 from Kawempe National Referral Hospital. The demographic characteristics of the participants are summarised in Table 1.

**Table 1:** Demographic characteristics of the participants from Kawempe and Mbale RRHs.

Characteristics	TOTAL 34
**Sex**	
Male	14
Female	20
**Professional experience**	
< 5 Years	10
5- 10 years	16
>10 years	8
**Cadre**	
Anaesthesiologists/ anaesthetic provider s	1
Obstetricians	6
Residents in Obstetrics and Gynaecology	8
Midwives	9
Interns	8
Medical officers	2

### Thematic analysis

This section presents findings structured around four primary themes: initial reactions when faced with a pregnant woman with SCD, social, cultural and religious issues affecting care, challenges within the healthcare system and strategies to improve care. Each theme is further divided into subthemes, with a focus on the corresponding levels of the BEF as shown in Table 2.

**Table 2:** Themes and subthemes corresponding with different levels of the Behavioural Ecological Framework examining healthcare access and navigation.

Level	Theme	Subtheme
Individual level	Initial reactions	Knowledge gap, perceived complexity and awareness
		Fear, anxiety and risk perception
		Emotional/psychological distress
Community level	Cultural, social and religious influences on care	Negative cultural beliefs
		Delay due to religious influence
		Lack of Social support systems
Health system level factors	Challenges in healthcare delivery	Limited ICU Space
		Diagnostic delays
		Limited resources (blood and medications) & Ethical dilemmas
		Lack of specialized services
		Limited Interdisciplinary collaborations
		High patient load
Policy level	Standardization of care	Lack of guidelines and protocols for managing SCD in pregnancy
		Inadequate formal/refresher training
Recommendations		Training and Refresher courses, guidelines, patient education and empowerment, psychosocial support and advocacy for more healthcare resources

### Individual level

#### Knowledge and awareness.

Healthcare providers demonstrated varied of understanding of SCD, with many demonstrating foundational knowledge of its implications in pregnancy. Most participants acknowledged SCD as a significant maternal health concern in Uganda, and expressed several recurring reactions when managing affected patients. Providers reported limited exposure to SCD management in pregnancy during formal training, resulting in knowledge gaps regarding tailored management strategies. This was observed by midwives and intern doctors, who emphasised the need for continuous professional development and training on the latest management protocols. A midwife from KNRH emphasized, “*We know the basics, but more training on the unique challenges faced by pregnant women with SCD could help us provide better care (HW10MK)”.*

#### Fear, anxiety and perception of risks.

Many providers demonstrated a general awareness of the increased maternal and fetal risks associated with SCD in pregnancy, including increased morbidity and mortality. However, perceptions of risk severity varied across cadres and study sites. At KNRH, all pregnant women admitted with SCD crises or for delivery were managed in the ICU with many undergoing Caesarean section irrespective of indication. In Mbale, the patients were admitted to antenatal wards but similarly delivered by caesarean section as standard protocol. Anaesthesiologists expressed concern regarding pain management and labour complications while midwives prioritised maternal and fetal monitoring. As one midwife remarked, *“I even fear to work on them (pregnant women with SCD), they are fragile, in pain, always pale. I don't want to work on them when I'm alone”* ***(HW10, MK)***

#### Emotional and psychological distress.

Beyond fear and anxiety, healthcare providers highlighted the emotional toll of caring for pregnant women with SCD. Many expressed empathy and concern, reflecting on the emotional distress experienced by their patients. A resident in Obstetrics and Gynaecology from Kawempe noted, ‘*You feel emotionally drained, especially if, after doing your best, you lose the patient’* ***(HW17, DK).***

Providers recognised that pregnant women with SCD often experience heightened anxiety and stress due to their condition, prompting some providers to adopt a more compassionate approach that integrates, psychological support with medical care. A midwife from Mbale stated, “*We try to reassure them (pregnant women with SCD) and even ourselves, to help them understand their condition and what to expect. It’s important for mental well-being. We even pray with them, and now we involve some expert mothers who have had the same journey.”*

### Community level

#### Cultural, social and religious influences.

The BEF posits that healthcare access and navigation are influenced by multiple community-level factors that interact dynamically. These include community resources, cultural norms and societal values regarding health that influence behaviour and utilization of health care services. The results of this study underscore the intricate interplay of cultural beliefs, religious influences, and social support systems in the management of pregnant women with sickle cell disease

Cultural beliefs significantly influence the perception and management of SCD in pregnancy. Health workers reported that many patients hold traditional views, including beliefs that SCD is a curse or a result of witchcraft, which can delay care seeking. *They believed someone bewitched her because she always lost her pregnancies* ***(HW11, MK).*** Moreover, cultural practices often dictate how women manage their health during pregnancy. A midwife from Mbale shared an example, noting, *“I had a patient who refused to take her prescribed medication. Women say they were told not to take iron tablets or injections They fear it harms the baby. This is common; they trust their culture more than the medical system.”* ***(HW6MM)****.*

Additionally, religious beliefs played a crucial role in the management of pregnant women with SCD. Many patients turn to their faith for comfort and guidance, which can provide emotional support, but sometimes hinder acceptance of medical interventions. A doctor from Kawempe explained that some refused blood transfusions or pain management due to religious beliefs. “*She belonged to Jehovah's Witnesses; she refused blood transfusion, complicating care”* **(HW17DK).** Health workers reported instances where religious leaders influenced patients’ decisions regarding their health. In some cases, religious doctrines were cited as reasons for avoiding certain medical treatments, particularly those involving blood and surgery. A midwife recounted a situation where a patient’s pastor was the one to decide whether the patient should have a caesarean section or not. “*The pastor was making decisions regarding the woman's caesarean section, delaying critical medical care”.* ***(HW9, MK).***

Conversely, some health workers acknowledged that active participation in religious communities often provided emotional and social support that positively impacted women’s health. Similarly, the lack of social support systems emerged as a critical theme affecting the management of pregnant women with SCD. Health workers consistently reported that many patients experience significant social isolation, lacking partner, family, friends, and community support.. A midwife from Mbale highlighted this, stating, ‘*Most of these girls come alone. No partner, no parent. It's heartbreaking’* ***(HW7, MM).*** This isolation makes it difficult for them to cope with the demands of pregnancy and SCD.” Socioeconomic constraints limit access to necessary healthcare services, including regular check-ups and pain management.

#### Health systems challenges.

Accessibility of healthcare facilities, availability of services, healthcare policies, and the quality of care provided are essential factors at this level. Organizational culture and practices, such as appointment scheduling, waiting times, and availability of supplies, can either enable or hinder care. Out-of-pocket costs and the complexity of the health care financing affect healthcare services delivery. This level was analyzed through both the BEF and the use of the WHO health system building blocks (WHO, 2007). The application of the WHO health system building blocks framework to the qualitative findings provides a comprehensive lens to understand the multifaceted barriers faced by health workers in managing pregnant women with SCD. The analysis revealed issues related to service delivery, health workforce, access to essential medicines, health information, financing, and leadership and governance.

#### Service delivery.

Service delivery was significantly hampered by *limited Intensive Care Unit (ICU) and High Dependency Unit (HDU) space*, which constrained the ability to manage critically ill pregnant women with SCD. This inadequacy undermines the provision of timely, appropriate care during obstetric emergencies, a core principle of effective service delivery (WHO, 2007).’ *By the time we transfer them, it’s late. We don’t always have quick ICU access for these mothers* ***(HW15 DK),*** stated one of the doctors in Kawempe.

Furthermore, the *lack of specialised services* for high-risk obstetric patients, such as those with SCD, reflects systemic gaps in the organisation and coordination of care. One of the doctors from Mbale observed, *‘Mothers with sickle cell need specialized antenatal services* ***(HW8, DM).*** Additionally, *diagnostic delays* due to equipment shortages or long turnaround times reduced the efficiency of the health system in responding to complications such as infections or thrombotic events, both of which are common in SCD pregnancy. These delays potentially contribute to increased maternal and perinatal morbidity and mortality.

Moreover, *high patient loads* were frequently mentioned, reflecting insufficient staffing relative to patient demand. This increases burnout and compromises the quality of care and patient safety. A midwife shared, “*With so many patients to attend to during High-Risk Clinics, it’s difficult to give each one the attention they deserve, especially those with complex conditions like SCD*.” A doctor from Kawempe stated: *“With over 200 patients to see per high-risk clinic day, I sometimes feel I cannot give each one the attention they deserve. This is especially true for those with SCD, who often require more time.”*

Additionally, the *lack of interdisciplinary collaboration*—for example, among obstetricians, haematologists, anaesthetists, and nurses—was a barrier to comprehensive and coordinated care, essential in managing complex cases like SCD in pregnancy. *Sometimes we forget to involve internal medicine or hematology because we don’t have them available. We miss important assessments that way* ***(HW2 DM)*** lamented one of the doctors from Mbale. *“If we could have regular meetings with haematologists and neonatologists, we could discuss our patients’ cases and develop better management plans together.* Providers reported that the limited availability of these specialists and NICUs often resulted in delays in care, which posed serious implications for maternal and fetal health. Additionally, the referral process, particularly from Kawempe to Mulago Specialized Women’s and Neonatal Hospital, was described as cumbersome, with communication gaps and financial burdens to patients. Most participants noted that patients usually got lost along the pathway after referrals because, upon discharge, they are told to continue ANC at Kawempe, so there is a lot of back and forth, confusing both patients and health care providers. Health workers highlighted frequent *stockouts of essential medications*, such as opioids for pain management, as well as thrombolytics like low molecular heparin, like Clexane and blood products. This undermines the ability to provide evidence-based care and exposes patients to avoidable suffering and complications. *Lack of medications* was particularly critical in managing vaso-occlusive crises and preventing infections, which are common and life-threatening in SCD. *“When we do not have the necessary medications or blood products, it is heartbreaking and very frustrating. We know what these patients need, but we cannot provide it”* stated one of the doctors from Kawempe. The *pain management dilemmas* reported by staff were partly due to these stockouts and partly due to the absence of clinical guidelines. Without clear protocols, providers were hesitant to administer strong analgesics, leading to suboptimal pain control and potential ethical concerns. *She was prescribed medications which weren't available in the pharmacy. She couldn't afford them herself* ***(HW8, DM)***

The study revealed an implicit challenge in health information systems, as a *lack of national guidelines* and poor access to evidence-based protocols limit the availability of reliable clinical information at the point of care. This gap hinders decision-making and reduces standardisation of care across facilities, as noted by the World Health Organisation. *Without established guidelines, we often rely on our personal experiences or what we have learned informally. This can lead to inconsistencies in care. You see like how we have preeclampsia guidelines and everyone knows, even without the doctor prescribing, the midwives already know how to treat, for SCD it is total confusion* ***(HW3 MFGD).***

#### Strategies for improved care.

Despite the challenges identified, participants also shared various strategies they believed could enhance the care provided to pregnant women with SCD. One key strategy mentioned was the establishment of an institutional protocol in Kawempe, where all SCD patients are admitted and managed in ICU/HDU by multidisciplinary care team to address the complex needs of these patients. Providers emphasized the importance of collaboration between obstetricians, anaesthesiologists and other specialists to ensure comprehensive care. *If we could have regular meetings with haematologists and neonatologists, we could discuss our patients’ cases and develop better management plans together (****HW3 MFGD).***

Additionally, the need for ongoing training and education for healthcare providers was a prominent theme. Participants expressed a desire for more targeted educational programs focusing on SCD management during pregnancy. This could include workshops, online courses, and simulation training to enhance clinical skills and knowledge. Providers believed that increased awareness and understanding of SCD would lead to better patient care and improved confidence in managing complex cases. Empowering patients with knowledge was seen as a way to enhance their engagement in care and improve adherence to recommendations. Additionally, participants highlighted the potential benefits of community education and outreach programs aimed at raising awareness about SCD and its implications during pregnancy. They believed that increasing public knowledge could help reduce stigma and encourage more women to seek care early in their pregnancies. One midwife from Mbale stated *‘’ You know like, how we have Preeclampsia brochure and PPH talking points for communities, one for SCD in pregnancy would also be very helpful during our health education talks in ANC and also outreaches.”* Providers also advocated for increased funding and support for healthcare facilities to ensure the availability of essential supplies and medications. Participants acknowledged that addressing these systemic issues would require advocacy at both institutional and governmental levels. Finally, the team re-echoed the necessity of developing standardized guidelines for the management of pregnancies complicated by SCD. They suggested that these guidelines should be evidence-based and tailored to the local context, considering the unique challenges faced by healthcare providers in their settings. The involvement of multidisciplinary teams in the guideline development process was emphasized as essential for ensuring comprehensive care. The introduction of standardized protocols for managing SCD in pregnancy was seen as a critical step toward improving care delivery.

## Discussion

This study provides vital insights into the complexities of managing SCD among pregnant women in a resource-limited setting. The findings highlight several key themes that reflect the multifaceted nature of SCD management and the critical need for comprehensive approaches that address both clinical and systemic issues.

### Individual level

The first theme, initial reactions, reveals varying levels of knowledge and awareness among healthcare providers regarding the disease. This aligns with previous studies indicating that healthcare professionals often possess inconsistent knowledge about SCD, which can significantly impact patient care [7]. The subtheme of perception of risks is particularly pertinent, as providers’ understanding of the disease's complications can influence their clinical decisions and patient outcomes. A lack of awareness regarding the specific risks associated with SCD in pregnancy may lead to inadequate monitoring and management, potentially jeopardising maternal and fetal health [8,9].

Furthermore, the Lancet Haematology Commission paper on SCD (2024) emphasises the importance of education and training for healthcare providers to improve outcomes for patients with SCD [10]. This highlights a critical gap in the Ugandan healthcare system, where ongoing professional development and training in SCD management are essential. The variability in knowledge and awareness among providers underscores the need for standardised curricula and training programmes that address the complexities of SCD, particularly in the context of pregnancy.

### Community level

The healthcare providers’ perspectives and experiences in managing pregnant women with Sickle Cell Disease (SCD), focusing on the theme of community-level influences, highlighted the profound role of cultural beliefs, religious practices, social norms, and social support systems in shaping maternal healthcare experiences and outcomes among women with SCD.

### Cultural influences, religious and social influences

Negative cultural beliefs and traditional understandings of illness emerged as critical barriers to effective care. Healthcare providers reported that in many communities, SCD is still poorly understood and often attributed to supernatural causes or ancestral curses. This aligns with previous findings in Nigeria, where SCD is frequently interpreted through traditional lenses, affecting families’ care-seeking behaviour [11]. Cultural stigma can delay access to antenatal services, as some families may hide affected individuals or prioritise traditional remedies over biomedical care. Furthermore, certain communities hold fatalistic views about SCD, seeing it as a “death sentence” and discouraging affected women from becoming pregnant. These beliefs reinforce discriminatory attitudes and reduce community support for reproductive health services, contributing to the marginalisation of women with SCD during pregnancy. Similar observations have been documented in Nigeria, where socio-cultural myths deter appropriate health-seeking for women with hereditary conditions [12].

Religion played a dual role in shaping perceptions of SCD and maternal care. On one hand, religious leaders were often trusted sources of guidance and could encourage acceptance and support for pregnant women with SCD. On the other hand, some religious doctrines promoted the rejection of biomedical interventions in favour of faith healing. These findings echo those of Cotton et al [13], who found that adolescents with SCD often relied on religious explanations and spiritual interventions as coping mechanisms for complications of SCD.

In Uganda, where this study was conducted, religious affiliation is widespread, and spiritual interpretations of illness often coexist with biomedical views. Participants described instances where religious leaders influenced pregnant women’s decisions, including discouraging the use of contraception or discouraging antenatal visits in favour of faith-based interventions. While religious beliefs can foster hope and resilience, they may also conflict with evidence-based medical care, especially when not integrated into public health messaging

Social stigma surrounding SCD was widely reported. Health workers noted that affected women are often labelled as weak or unfit for motherhood, leading to isolation and diminished self-worth. This stigma influences marital prospects, family support, and women's willingness to disclose their condition, as observed in other contexts [14].

The study found that stigma extends to the health system, where healthcare providers, particularly in under-resourced settings, may hold misconceptions about SCD or show less empathy due to the perceived burden of care. This internalised bias within health facilities reinforces community-level stigma, ultimately affecting the quality of care delivered to pregnant women with SCD.

Despite these challenges, social support especially from family members and organised patient support groups, emerged as a protective factor. Pregnant women who had partners, families, or peer support groups were more likely to adhere to care regimens and attend antenatal appointments. This is consistent with previous research showing that family and community support can mitigate the psychological and physical burdens of chronic illness during pregnancy [15]. Participants also emphasised the importance of health education targeting both men and women in the community, noting that informed spouses and families were more likely to offer support. Encouragingly, in some settings, community health workers have played a pivotal role in bridging gaps between health facilities and families, increasing health literacy, and reducing stigma [15].

The findings underscore the complex interplay between cultural, religious, and social contexts in the management of pregnant women with SCD. Effective interventions must therefore be context-sensitive, engaging not just the health system but also community leaders, religious institutions, and social networks to promote awareness, reduce stigma, and improve outcomes. Integrating community-based health education with culturally competent care will be essential to addressing the multifaceted challenges experienced by this vulnerable population.

### Policy and healthcare delivery challenges

The need for evidence-based guidelines is evident, as existing literature supports the notion that standardised protocols can enhance the quality of care for patients with chronic diseases, including SCD [16,17]. The establishment of clinical guidelines that are specifically tailored to the needs of pregnant women with SCD could facilitate more consistent and effective management, ultimately improving maternal and fetal outcomes [18–20]. The absence of such guidelines in Uganda highlights a critical area for intervention, as the development and dissemination of evidence-based protocols would provide a framework for healthcare providers to deliver care that is both effective and aligned with best practices. The third theme, challenges in healthcare delivery, encompasses various subthemes that reflect the systemic issues faced by health providers. Resource limitations, including inadequate staffing, insufficient medical supplies, and lack of access to essential diagnostic tools, were frequently cited as barriers to effective care. This is consistent with findings from other studies that have identified similar challenges in low-resource settings, where healthcare providers often struggle to deliver quality care due to systemic constraints [17]. Referral challenges also emerged as a significant concern, with providers noting difficulties in accessing specialised care for patients with SCD. This is particularly relevant in the context of Uganda, where geographical barriers and limited healthcare infrastructure can impede timely referrals [21,22]. The importance of interdisciplinary collaborations in overcoming these challenges cannot be overstated. Collaborative approaches that involve multiple healthcare disciplines can enhance the management of complex cases, ensuring that patients receive comprehensive care that addresses both their medical and psychosocial needs [2,23]. Moreover, high patient loads and emotional and psychological distress among both patients and providers were highlighted as critical challenges. The burden of managing a chronic illness like SCD, particularly during pregnancy, can lead to significant psychological strain, which may further complicate care delivery [24]. Addressing the emotional and psychological needs of both patients and providers is essential for improving overall healthcare outcomes and fostering a supportive environment for care [17,24])] Advocacy for resources is another vital strategy, as increased funding and support for SCD programmes can help address the systemic challenges faced by healthcare providers and patients alike [16,25].

### Study strengths and limitations

The research team actively participates in the management of patients with SCD at the respective study sites and therefore, their experiences, beliefs and understanding might influence the study in certain aspects. Thus, to ensure rigor and validity, the team triangulated various data sources and methods (e.g., interviews, discussions, and reflective journals), enhancing the credibility of the study findings. Additionally, member checking and engaging participants in the validation of study findings were done to ensure that their perspectives were accurately represented.

This study was conducted at MRRH and KNRH, two high-volume public referral facilities that provide care for pregnant women with sickle cell disease in eastern and central Uganda. These facilities serve diverse urban, peri-urban, and rural populations and reflect typical public-sector referral settings in the region. The findings are applicable to similar resource-limited referral hospitals characterized by high patient volumes, constrained staffing, and limited access to specialized diagnostics. However, the study was conducted exclusively in public facilities where services are provided at no or minimal cost, and experiences may differ in private or faith-based health facilities.

Despite these limitations, the detailed contextual reporting and methodological rigor support the transferability of findings to comparable settings managing pregnancy in women with sickle cell disease.

## Conclusion

In exploring the experiences of health workers who manage pregnant women with SCD, the study identified several themes based on various health system building blocks and the different levels of the Behavioral Ecological Framework. Individual-level themes included knowledge gaps and awareness, fear and anxiety related to managing a pregnant woman with SCD, and emotional and psychological distress. Community-level influences consisted of religious beliefs, cultural beliefs, as well as myths and misconceptions. At the health systems level, key themes encompassed diagnostic challenges, shortages of critical supplies, lack of management guidelines, and the absence of multidisciplinary and specialized services.

## Recommendations

The study highlights the multifaceted challenges associated with caring for pregnant women with SCD in Uganda. The findings underscore the need for enhanced pre- and in-service training, the establishment of standardised context-specific guidelines, and community sensitisation campaigns about SCD. Addressing the systemic challenges in healthcare delivery will require a concerted effort from all stakeholders, including healthcare providers, policymakers, and patient advocacy groups. By prioritising these areas, it is possible to improve the quality of care for pregnant women with SCD and ultimately enhance health outcomes for this vulnerable population.
