# Supplementary material for: “Heartbreak and Frustration’’ experiences of healthcare providers caring for pregnant women with sickle cell disease in Uganda
Source: PLoS One. 2026 Aug 7;21(8):e0355018. doi: 10.1371/journal.pone.0355018 (PMC13450732; doi:10.1371/journal.pone.0355018)
Supplement: Supplementary file 1 [file pone.0355018.s003.docx]

 **MAKERERE**	UNIVERSITY

**P.O. Box 7072 Kampala-Uganda**

**Fax, 256414532591	Phone: +256-414-534361**

**Email: obsgyn@chs.mak.ac.ug**

#### **COLLEGE OF HEALTH SCIENCES**

**DEPARTMENT OF OBSTETRICS AND GYNAECOLOGY**

**09/05/2026**

Dear Academic Editor and Reviewers,

**Manuscript: “Heartbreak and Frustration” Experiences of Healthcare Providers Caring for Pregnant Women with Sickle Cell Disease in Uganda**

We are grateful for the careful review of our manuscript and for identifying citation-export and reference-formatting issues that required manual correction before the manuscript could proceed to production. We have undertaken a complete manual audit of the reference list and corrected the flagged references directly in the revised manuscript, with tracked changes retained for transparency.

Below we provide a point-by-point response to each comment raised.

**Point by point response**

| **No.** | **Editor/Reviewer comment** | **Response and revision made** |
| --- | --- | --- |
| 1 | Raw HTML markup (The Lancet Hematology) rendered visibly in Reference 3. | **Corrected.** The visible HTML tags around the journal title were removed, and the reference was reformatted in standard Vancouver style. The corrected citation now begins with the article title, followed by the journal title, year, volume, issue and page range, without visible markup. |
| 2 | The given name “Ifeanyi” was left unabbreviated in Reference 8, contrary to standard Vancouver conventions. | **Corrected**. The author names in Reference 8 were manually revised to Vancouver format. “Johnson Onyekpa Ifeanyi” was corrected to initials-based formatting, and the author string was cleaned so that all given names are appropriately abbreviated. |
| 3 | Academic credentials (e.g., BSN, RNM, PHN, FWACN, STTI) were incorrectly parsed as author name components in Reference 14. | **Corrected.** Academic and professional credentials were removed from the author field in Reference 14. The duplicated/erroneously parsed author names were also cleaned, leaving the correct author list and journal citation details in Vancouver style. |
| 4 | Journal-section metadata (“Obstet G, Acc O”) was erroneously listed as author names in Reference 23. | **Corrected.** The journal-section metadata was removed from the author field in Reference 23. The reference was manually revised so that only the actual authors remain in the author position, followed by the article title and journal details. |
| 5 | A complete, manual citation audit for each referenced source is required to fix systematic export errors before the manuscript proceeds to production. | **Completed.** Beyond the four references specifically flagged, the full reference list was manually audited for consistency with Vancouver style and production readiness. Additional corrections were made where needed, including journal-title abbreviation cleanup, page/article-number formatting, removal or standardization of inconsistent “[Internet]/Available from” export text, spacing and punctuation cleanup, and correction of minor author-name formatting issues. All such corrections were made directly in the manuscript and retained as tracked changes. |

**Summary of revision**

- The four references explicitly flagged by the editor/reviewer were corrected manually.
- The full reference list was reviewed for systematic export and formatting errors.
- Additional citation-formatting issues identified during the audit were corrected.
- All edits were made directly in the revised manuscript and preserved as tracked changes.

We appreciate the opportunity to revise the manuscript and trust that the corrected reference list now addresses the citation-formatting concerns raised during editorial review.

Sincerely,

Jackline Akello (On behalf of the authors)

Lecturer, Makerere University, College of Health Sciences, P.O. Box 7072

Tel: +256779 755663/+256772671340
