# Supplementary material for: “Heartbreak and Frustration’’ experiences of healthcare providers caring for pregnant women with sickle cell disease in Uganda
Source: PLoS One. 2026 Aug 7;21(8):e0355018. doi: 10.1371/journal.pone.0355018 (PMC13450732; doi:10.1371/journal.pone.0355018)
Supplement: Peer review history

# Peer review: Decision Letter 0

**DOI:** 10.1371/journal.pone.0355018.r001

**Authors:** Rehab Al-Ansari

12 Jan 2026

PONE-D-25-50130

“Heartbreak and Frustration’’ Experiences of Healthcare Providers Caring for Pregnant Women with Sickle Cell Disease in Uganda”

PLOS One

Dear Dr. Jackline Akello,

Thank you for submitting your manuscript to PLOS ONE. After careful consideration, we feel that it has merit but does not fully meet PLOS ONE’s publication criteria as it currently stands. Therefore, we invite you to submit a revised version of the manuscript that addresses the points raised during the review process.

==============================-->Dear author

Despite having a limited sample size, the study is distinctive and could be useful for future research. Nevertheless, small oversights need be fixed as fowling:

- When utilizing a shortened form for the first time, please write its complete name, such as (IDI).

- Include the study's small sample size as a limitation.

==============================

Please submit your revised manuscript by Feb 26 2026 11:59PM. If you will need more time than this to complete your revisions, please reply to this message or contact the journal office at plosone@plos.org. When you're ready to submit your revision, log on to https://www.editorialmanager.com/pone/ and select the 'Submissions Needing Revision' folder to locate your manuscript file.

Please include the following items when submitting your revised manuscript:

- A letter that responds to each point raised by the academic editor and reviewer(s). You should upload this letter as a separate file labeled 'Response to Reviewers'.
- A marked-up copy of your manuscript that highlights changes made to the original version. You should upload this as a separate file labeled 'Revised Manuscript with Track Changes'.
- An unmarked version of your revised paper without tracked changes. You should upload this as a separate file labeled 'Manuscript'.

-->If you would like to make changes to your financial disclosure, please include your updated statement in your cover letter. Guidelines for resubmitting your figure files are available below the reviewer comments at the end of this letter.

If applicable, we recommend that you deposit your laboratory protocols in protocols.io to enhance the reproducibility of your results. Protocols.io assigns your protocol its own identifier (DOI) so that it can be cited independently in the future. For instructions see: https://journals.plos.org/plosone/s/submission-guidelines#loc-laboratory-protocols. Additionally, PLOS ONE offers an option for publishing peer-reviewed Lab Protocol articles, which describe protocols hosted on protocols.io. Read more information on sharing protocols at https://plos.org/protocols?utm_medium=editorial-email&utm_source=authorletters&utm_campaign=protocols.

We look forward to receiving your revised manuscript.

Kind regards,

Rehab Al-Ansari

Academic Editor

PLOS One

Journal Requirements:

When submitting your revision, we need you to address these additional requirements.

1. Please ensure that your manuscript meets PLOS ONE's style requirements, including those for file naming. The PLOS ONE style templates can be found at

https://journals.plos.org/plosone/s/file?id=wjVg/PLOSOne_formatting_sample_main_body.pdf and

https://journals.plos.org/plosone/s/file?id=ba62/PLOSOne_formatting_sample_title_authors_affiliations.pdf

2. Thank you for stating the following financial disclosure:

“Research reported in this publication is funded by The National Heart, Lung, And Blood Institute of the National Institutes of Health, Award Number D43TW012466 through the Enhancing Research Capacity for Sickle Cell Disease and related NCDs across the Lifespan in Uganda (Enrich Project) D43TWO12466-01.  The content is solely the responsibility of the authors and does not necessarily represent the official views of the National Institutes of Health.”

Please state what role the funders took in the study. If the funders had no role, please state: "The funders had no role in study design, data collection and analysis, decision to publish, or preparation of the manuscript."

If this statement is not correct you must amend it as needed.

Please include this amended Role of Funder statement in your cover letter; we will change the online submission form on your behalf.

3. We note that you have indicated that there are restrictions to data sharing for this study. For studies involving human research participant data or other sensitive data, we encourage authors to share de-identified or anonymized data. However, when data cannot be publicly shared for ethical reasons, we allow authors to make their data sets available upon request. For information on unacceptable data access restrictions, please see http://journals.plos.org/plosone/s/data-availability#loc-unacceptable-data-access-restrictions.

Before we proceed with your manuscript, please address the following prompts:

a) If there are ethical or legal restrictions on sharing a de-identified data set, please explain them in detail (e.g., data contain potentially identifying or sensitive patient information, data are owned by a third-party organization, etc.) and who has imposed them (e.g., a Research Ethics Committee or Institutional Review Board, etc.). Please also provide contact information for a data access committee, ethics committee, or other institutional body to which data requests may be sent.

b) If there are no restrictions, please upload the minimal anonymized data set necessary to replicate your study findings to a stable, public repository and provide us with the relevant URLs, DOIs, or accession numbers. Please see http://www.bmj.com/content/340/bmj.c181.long for guidelines on how to de-identify and prepare clinical data for publication. For a list of recommended repositories, please see https://journals.plos.org/plosone/s/recommended-repositories. You also have the option of uploading the data as Supporting Information files, but we would recommend depositing data directly to a data repository if possible.

Please update your Data Availability statement in the submission form accordingly.

4. When completing the data availability statement of the submission form, you indicated that you will make your data available on acceptance. We strongly recommend all authors decide on a data sharing plan before acceptance, as the process can be lengthy and hold up publication timelines. Please note that, though access restrictions are acceptable now, your entire data will need to be made freely accessible if your manuscript is accepted for publication. This policy applies to all data except where public deposition would breach compliance with the protocol approved by your research ethics board. If you are unable to adhere to our open data policy, please kindly revise your statement to explain your reasoning and we will seek the editor's input on an exemption. Please be assured that, once you have provided your new statement, the assessment of your exemption will not hold up the peer review process.

5. If the reviewer comments include a recommendation to cite specific previously published works, please review and evaluate these publications to determine whether they are relevant and should be cited. There is no requirement to cite these works unless the editor has indicated otherwise.

6. Please review your reference list to ensure that it is complete and correct. If you have cited papers that have been retracted, please include the rationale for doing so in the manuscript text, or remove these references and replace them with relevant current references. Any changes to the reference list should be mentioned in the rebuttal letter that accompanies your revised manuscript. If you need to cite a retracted article, indicate the article’s retracted status in the References list and also include a citation and full reference for the retraction notice.

Additional Editor Comments:

Dear author

Despite having a limited sample size, the study is distinctive and could be useful for future research. Nevertheless, small oversights need be fixed as fowling:

- When utilizing a shortened form for the first time, please write its complete name, such as (IDI).

- Include the study's small sample size as a limitation.

best regards

[Note: HTML markup is below. Please do not edit.]

Reviewers' comments:

Reviewer's Responses to Questions

**Comments to the Author**

1. Is the manuscript technically sound, and do the data support the conclusions?

The manuscript must describe a technically sound piece of scientific research with data that supports the conclusions. Experiments must have been conducted rigorously, with appropriate controls, replication, and sample sizes. The conclusions must be drawn appropriately based on the data presented.

Reviewer #1: Yes

Reviewer #2: Yes

**********

2. Has the statistical analysis been performed appropriately and rigorously?

Reviewer #1: No

Reviewer #2: I Don't Know

**********

3. Have the authors made all data underlying the findings in their manuscript fully available?

The PLOS Data policy requires authors to make all data underlying the findings described in their manuscript fully available without restriction, with rare exception (please refer to the Data Availability Statement in the manuscript PDF file). The data should be provided as part of the manuscript or its supporting information, or deposited to a public repository. For example, in addition to summary statistics, the data points behind means, medians and variance measures should be available. If there are restrictions on publicly sharing data—e.g. participant privacy or use of data from a third party—those must be specified.

Reviewer #1: Yes

Reviewer #2: Yes

**********

4. Is the manuscript presented in an intelligible fashion and written in standard English?

PLOS ONE does not copyedit accepted manuscripts, so the language in submitted articles must be clear, correct, and unambiguous. Any typographical or grammatical errors should be corrected at revision, so please note any specific errors here.

Reviewer #1: No

Reviewer #2: Yes

**********

5. Review Comments to the Author

Please use the space provided to explain your answers to the questions above. You may also include additional comments for the author, including concerns about dual publication, research ethics, or publication ethics. (Please upload your review as an attachment if it exceeds 20,000 characters)

Reviewer #1: Comments

1. Methods — Sampling and saturation: the manuscript states 34 participants (10 IDIs + 2 FGDs at KNRH; 4 IDIs + 2 FGDs at MRRH) but the description of participant selection, purposive sampling strategy, and how saturation was assessed is brief. Please clarify inclusion numbers by cadre per interview type, rationale for FGD sizes, how and when saturation was determined, and whether any potential voices (e.g., haematologists, NICU nurses) were underrepresented.

2. Data analysis transparency and rigor: the manuscript says thematic analysis was used but lacks procedural detail. Provide: who coded transcripts, whether coding was inductive/deductive, codebook development, use of software (if any), double-coding or inter-coder reliability procedures, and examples of how themes were derived from codes. Describe reflexivity procedures more concretely (how biases were managed).

3. Ethical details and participant confidentiality: the ethics approvals and consent are stated; add explicit information on confidentiality protections, how recordings/transcripts were stored, and whether participants received any compensation. Also clarify exclusion of minors (already noted) and whether any participants declined to participate..

4. Transferability/limitations: the limitations section notes researcher involvement and triangulation but should discuss transferability/generalizability more directly — e.g., how representative are these two referral hospitals of other Ugandan or regional settings, and how might site-specific practices (admission to ICU for all SCD deliveries at KNRH) affect findings.

5. Citations and some factual claims: multiple prevalence and service-use statistics are cited but a few claims (e.g., “KNRH receives about 140 pregnant women with SCD annually”; “MRRH receives two to five new SCD clinic cases daily”) would benefit from a source or internal registry reference or should be qualified as the sites’ estimates.

Minor concerns

1. Title punctuation: remove the extra closing quotation mark at the end of the title string. Line 1 & 2

2. Grammar and typos: several small errors (e.g., “Easten” → “Eastern”; “hematic analysis” → “thematic analysis”; inconsistent spacing and occasional run-on sentences). A careful language edit is recommended. Line 80 & 43

3. Table 1 formatting: combine broken rows and ensure each cell is single line per journal table rules

Reviewer #2: A well-detailed study narrative utilizing the method of qualitative research for in-depth interviews and focus group discussions of diverse healthcare practitioners who care for pregnant women with sickle cell disease (SCD).

The study’s indication that existing research focuses largely on epidemiology and clinical outcomes of sickle cell disease in pregnant women, without much research on the health care practitioners' perspectives for women in limited resource settings, is greatly applauded.

The study's use of a wide array of healthcare practitioners for participation in Group discussions provided an opportunity for variation of perspectives. Such healthcare practitioners as obstetricians and midwives, doctors, residents and medical officers gave consents for participation, and purposive sampling was utilized to ensure representation.

The aim of this study reveal the lack of a comprehensive sickle cell disease management protocol. And it highlights the healthcare practitioners' role in managing sickle cell disease in pregnant women as a very crucial role, with emphasis on identifying the opportunities to help strengthen care delivery practices.

Following data collection through interviews and discussions, thematic analysis was conducted to identify key themes and patterns. Several themes were identified at various levels, with highlighted suggestions for each identified theme.

Making free educational courses available to identify and bridge the knowledge gaps vital for efficiently managing pregnant women with sickle cell disease can help address the individual-level fears and anxieties. In addition, holding regular roundtable or Zoom discussions will help encourage healthcare practitioners to share their views and perspectives on management plans, setbacks, and triumphs, thereby supporting their peers who may be experiencing similar management challenges for this vulnerable population.

On the community level theme, inviting healthcare practitioners as guest speakers to community gatherings or church or cultural gatherings where basic knowledge education of sickle cell disease is disclosed to community members can help dispel myths from traditions, such as the belief that sickle cell disease is a curse or result of witchcraft.

On the health system level, developing a health system that promotes interdisciplinary collaboration and reduces communication gaps creates a clearer protocol for sickle cell disease management and a better standardization of care across facilities where pregnant women with sickle cell disease are referred for treatment.

Finally, an excellent recommendation was highlighted in the discussion phase of the study, which reminds us of the multifaceted challenges associated with caring for this vulnerable population in Uganda. It further discloses that addressing these challenges requires a group effort of all stakeholders, such as healthcare providers, policymakers, and patient advocacy groups. This effort will help improve the chances of a better health outcome for this vulnerable population with limited resources.

Thank you for the invitation to review this very important study. It is highly recommended for publication

**********

6. PLOS authors have the option to publish the peer review history of their article (what does this mean?). If published, this will include your full peer review and any attached files.

If you choose “no”, your identity will remain anonymous but your review may still be made public.

**Do you want your identity to be public for this peer review?**  For information about this choice, including consent withdrawal, please see our Privacy Policy.

Reviewer #1: No

Reviewer #2: **Yes:** Nonye Tochi Aghanya MSc, RN, FNP-C

**********

[NOTE: If reviewer comments were submitted as an attachment file, they will be attached to this email and accessible via the submission site. Please log into your account, locate the manuscript record, and check for the action link "View Attachments". If this link does not appear, there are no attachment files.]

To ensure your figures meet our technical requirements, please review our figure guidelines: https://journals.plos.org/plosone/s/figures

You may also use PLOS’s free figure tool, NAAS, to help you prepare publication quality figures: https://journals.plos.org/plosone/s/figures#loc-tools-for-figure-preparation.

NAAS will assess whether your figures meet our technical requirements by comparing each figure against our figure specifications.

# Author comment: Author response to Decision Letter 1

**DOI:** 10.1371/journal.pone.0355018.r002

19 Jan 2026

Comment Response to the reviewers

Academic Editor's comments

Please ensure that your manuscript meets PLOS ONE's style requirements, including those for file naming. The PLOS ONE style templates can be found at

https://journals.plos.org/plosone/s/file?id=wjVg/PLOSOne_formatting_sample_main_body.pdf and

https://journals.plos.org/plosone/s/file?id=ba62/PLOSOne_formatting_sample_title_authors_affiliations.pdf

The title page and author affiliations have been revised in line with the journal guidelines. The main body of the manuscript has also been edited to align with the formatting guidelines.

Thank you for stating the following financial disclosure:

“Research reported in this publication is funded by The National Heart, Lung, And Blood Institute of the National Institutes of Health, Award Number D43TW012466 through the Enhancing Research Capacity for Sickle Cell Disease and related NCDs across the Lifespan in Uganda (Enrich Project) D43TWO12466-01. The content is solely the responsibility of the authors and does not necessarily represent the official views of the National Institutes of Health.”

Please state what role the funders took in the study. If the funders had no role, please state: "The funders had no role in study design, data collection and analysis, decision to publish, or preparation of the manuscript."

If this statement is not correct, you must amend it as needed.

Please include this amended Role of Funder statement in your cover letter; we will change the online submission form on your behalf.

Thank you for this feedback. The financial disclosure has been updated to include the statement “The funders had no role in study design, data collection and analysis, decision to publish, or preparation of the manuscript."

We note that you have indicated that there are restrictions to data sharing for this study. For studies involving human research participant data or other sensitive data, we encourage authors to share de-identified or anonymized data. However, when data cannot be publicly shared for ethical reasons, we allow authors to make their data sets available upon request. For information on unacceptable data access restrictions, please see http://journals.plos.org/plosone/s/data-availability#loc-unacceptable-data-access-restrictions.

Before we proceed with your manuscript, please address the following prompts:

a) If there are ethical or legal restrictions on sharing a de-identified data set, please explain them in detail (e.g., data contain potentially identifying or sensitive patient information, data are owned by a third-party organization, etc.) and who has imposed them (e.g., a Research Ethics Committee or Institutional Review Board, etc.). Please also provide contact information for a data access committee, ethics committee, or other institutional body to which data requests may be sent.

b) If there are no restrictions, please upload the minimal anonymized data set necessary to replicate your study findings to a stable, public repository and provide us with the relevant URLs, DOIs, or accession numbers. Please see http://www.bmj.com/content/340/bmj.c181.long for guidelines on how to de-identify and prepare clinical data for publication. For a list of recommended repositories, please see https://journals.plos.org/plosone/s/recommended-repositories. You also have the option of uploading the data as Supporting Information files, but we would recommend depositing data directly to a data repository if possible.

Please update your Data Availability statement in the submission form accordingly.

We regret the mix-up regarding the data availability. The data has been uploaded with open access in a public repository via the link. https://doi.org/10.7910/DVN/9GMSRE

When completing the data availability statement of the submission form, you indicated that you will make your data available on acceptance. We strongly recommend all authors decide on a data sharing plan before acceptance, as the process can be lengthy and hold up publication timelines. Please note that, though access restrictions are acceptable now, your entire data will need to be made freely accessible if your manuscript is accepted for publication. This policy applies to all data except where public deposition would breach compliance with the protocol approved by your research ethics board. If you are unable to adhere to our open data policy, please kindly revise your statement to explain your reasoning and we will seek the editor's input on an exemption. Please be assured that, once you have provided your new statement, the assessment of your exemption will not hold up the peer review process. The dataset has been made available and a link to the dataset shared

If the reviewer comments include a recommendation to cite specific previously published works, please review and evaluate these publications to determine whether they are relevant and should be cited. There is no requirement to cite these works unless the editor has indicated otherwise.

There have been no further requests for additional citations.

Please review your reference list to ensure that it is complete and correct. If you have cited papers that have been retracted, please include the rationale for doing so in the manuscript text, or remove these references and replace them with relevant current references. Any changes to the reference list should be mentioned in the rebuttal letter that accompanies your revised manuscript. If you need to cite a retracted article, indicate the article’s retracted status in the References list and also include a citation and full reference for the retraction notice. While no reviewer has made a comment on the references, we have reviewed and cited appropriately using Mendeley as a reference manager using Vancouver style as recommended by the journal

Reviewer 1 comments

1. Methods — Sampling and saturation: the manuscript states 34 participants (10 IDIs + 2 FGDs at KNRH; 4 IDIs + 2 FGDs at MRRH) but the description of participant selection, purposive sampling strategy, and how saturation was assessed is brief. Please clarify inclusion numbers by cadre per interview type, rationale for FGD sizes, how and when saturation was determined, and whether any potential voices (e.g., haematologists, NICU nurses) were underrepresented. Thank you for raising this critical issue. The section on study procedure and sampling has been enhanced to detail the justification for the purposive sampling. The section on data analysis has also been updated to detail how saturation was arrived at.

Please refer to line 142-153, 170-177

2. Data analysis transparency and rigor: the manuscript says thematic analysis was used but lacks procedural detail. Provide: who coded transcripts, whether coding was inductive/deductive, codebook development, use of software (if any), double-coding or inter-coder reliability procedures, and examples of how themes were derived from codes. Describe reflexivity procedures more concretely (how biases were managed). The rigor and procedural details have been enhanced to capture the analysis processes including how code book was generated and the thematic analysis plan.

3. Ethical details and participant confidentiality: the ethics approvals and consent are stated; add explicit information on confidentiality protections, how recordings/transcripts were stored, and whether participants received any compensation. Also clarify exclusion of minors (already noted) and whether any participants declined to participate. The section on ethical standards and approvals has been detailed to include participants confidentiality, compensation and handling of transcripts.

Please refer to lines 134-141

4. Transferability/limitations: the limitations section notes researcher involvement and triangulation but should discuss transferability/generalizability more directly — e.g., how representative are these two referral hospitals of other Ugandan or regional settings, and how might site-specific practices (admission to ICU for all SCD deliveries at KNRH) affect findings. The section on study strengths and limitations has been enhanced to address concerns of transferability /generalizability of the study.

Please refer to line 189- 195.

5. Citations and some factual claims: multiple prevalence and service-use statistics are cited but a few claims (e.g., “KNRH receives about 140 pregnant women with SCD annually”; “MRRH receives two to five new SCD clinic cases daily”) would benefit from a source or internal registry reference or should be qualified as the sites’ estimates.

This has been revised to show that the statistics used are site estimates and not published data.

Please refer to line 109-115

6. Title punctuation: remove the extra closing quotation mark at the end of the title string. Line 1 & 2 This has been removed.

7. Grammar and typos: several small errors (e.g., “Easten” → “Eastern”; “hematic analysis” → “thematic analysis”; inconsistent spacing and occasional run-on sentences). A careful language edit is recommended. Line 80 & 43 The entire document has been proof read and edited to ensure grammatical errors are corrected.

8. Table 1 formatting: combine broken rows and ensure each cell is single line per journal table rules

Corrected on page 8

Reviewer 2 comments

A well-detailed study narrative utilizing the method of qualitative research for in-depth interviews and focus group discussions of diverse healthcare practitioners who care for pregnant women with sickle cell disease (SCD). Thank you for the detailed review and appreciation of the study. No specific comment to be addressed but we note the specific action points suggested as per theme.

# Peer review: Decision Letter 1

**DOI:** 10.1371/journal.pone.0355018.r003

**Authors:** Rehab Al-Ansari

8 May 2026

PONE-D-25-50130R1

“Heartbreak and Frustration’’ Experiences of Healthcare Providers Caring for Pregnant Women with Sickle Cell Disease in Uganda”

PLOS One

Dear Dr. Akello,

Thank you for submitting your manuscript to PLOS ONE. After careful consideration, we feel that it has merit but does not fully meet PLOS ONE’s publication criteria as it currently stands. Therefore, we invite you to submit a revised version of the manuscript that addresses the points raised during the review process.

Please submit your revised manuscript by Jun 22 2026 11:59PM. If you will need more time than this to complete your revisions, please reply to this message or contact the journal office at plosone@plos.org. When you're ready to submit your revision, log on to https://www.editorialmanager.com/pone/ and select the 'Submissions Needing Revision' folder to locate your manuscript file.

Please include the following items when submitting your revised manuscript:

- A letter that responds to each point raised by the academic editor and reviewer(s). You should upload this letter as a separate file labeled 'Response to Reviewers'.
- A marked-up copy of your manuscript that highlights changes made to the original version. You should upload this as a separate file labeled 'Revised Manuscript with Track Changes'.
- An unmarked version of your revised paper without tracked changes. You should upload this as a separate file labeled 'Manuscript'.

If you would like to make changes to your financial disclosure, please include your updated statement in your cover letter. Guidelines for resubmitting your figure files are available below the reviewer comments at the end of this letter.

If applicable, we recommend that you deposit your laboratory protocols in protocols.io to enhance the reproducibility of your results. Protocols.io assigns your protocol its own identifier (DOI) so that it can be cited independently in the future. For instructions see: https://journals.plos.org/plosone/s/submission-guidelines#loc-laboratory-protocols. Additionally, PLOS ONE offers an option for publishing peer-reviewed Lab Protocol articles, which describe protocols hosted on protocols.io. Read more information on sharing protocols at https://plos.org/protocols?utm_medium=editorial-email&utm_source=authorletters&utm_campaign=protocols.

As the corresponding author, your ORCID iD is verified in the submission system and will appear in the published article. PLOS supports the use of ORCID, and we encourage all coauthors to register for an ORCID iD and use it as well. Please encourage your coauthors to verify their ORCID iD within the submission system before final acceptance, as unverified ORCID iDs will not appear in the published article. *Only*  the individual author can complete the verification step; PLOS staff *cannot*  verify ORCID iDs on behalf of authors.

We look forward to receiving your revised manuscript.

Kind regards,

Rehab Al-Ansari

Academic Editor

PLOS One

Journal Requirements:

1. If the reviewer comments include a recommendation to cite specific previously published works, please review and evaluate these publications to determine whether they are relevant and should be cited. There is no requirement to cite these works unless the editor has indicated otherwise.

2. Please review your reference list to ensure that it is complete and correct. If you have cited papers that have been retracted, please include the rationale for doing so in the manuscript text, or remove these references and replace them with relevant current references. Any changes to the reference list should be mentioned in the rebuttal letter that accompanies your revised manuscript. If you need to cite a retracted article, indicate the article’s retracted status in the References list and also include a citation and full reference for the retraction notice.

Additional Editor Comments:

Further revision is required, please indorse reviewer comment.

Reviewers' comments:

Reviewer's Responses to Questions

**Comments to the Author**

1. If the authors have adequately addressed your comments raised in a previous round of review and you feel that this manuscript is now acceptable for publication, you may indicate that here to bypass the “Comments to the Author” section, enter your conflict of interest statement in the “Confidential to Editor” section, and submit your "Accept" recommendation.

Reviewer #2: All comments have been addressed

Reviewer #3: All comments have been addressed

**********

2. Is the manuscript technically sound, and do the data support the conclusions?

The manuscript must describe a technically sound piece of scientific research with data that supports the conclusions. Experiments must have been conducted rigorously, with appropriate controls, replication, and sample sizes. The conclusions must be drawn appropriately based on the data presented.

Reviewer #2: Yes

Reviewer #3: Yes

**********

3. Has the statistical analysis been performed appropriately and rigorously?

Reviewer #2: I Don't Know

Reviewer #3: N/A

**********

4. Have the authors made all data underlying the findings in their manuscript fully available?

The PLOS Data policy requires authors to make all data underlying the findings described in their manuscript fully available without restriction, with rare exception (please refer to the Data Availability Statement in the manuscript PDF file). The data should be provided as part of the manuscript or its supporting information, or deposited to a public repository. For example, in addition to summary statistics, the data points behind means, medians and variance measures should be available. If there are restrictions on publicly sharing data—e.g. participant privacy or use of data from a third party—those must be specified.

Reviewer #2: Yes

Reviewer #3: Yes

**********

5. Is the manuscript presented in an intelligible fashion and written in standard English?

PLOS ONE does not copyedit accepted manuscripts, so the language in submitted articles must be clear, correct, and unambiguous. Any typographical or grammatical errors should be corrected at revision, so please note any specific errors here.

Reviewer #2: Yes

Reviewer #3: Yes

**********

6. Review Comments to the Author

Please use the space provided to explain your answers to the questions above. You may also include additional comments for the author, including concerns about dual publication, research ethics, or publication ethics. (Please upload your review as an attachment if it exceeds 20,000 characters)

Reviewer #2: Thank you for addressing my previous below concerns/recommendations by Including Strategies for Improved Care in the Revised manuscript.

"Making free educational courses available to identify and bridge the knowledge gaps vital for efficiently managing pregnant women with sickle cell disease can help address the individual-level fears and anxieties. In addition, holding regular roundtable or Zoom discussions will help encourage healthcare practitioners to share their views and perspectives on management plans, setbacks, and triumphs, thereby supporting their peers who may be experiencing similar management challenges for this vulnerable population.

On the community level theme, inviting healthcare practitioners as guest speakers to community gatherings or church or cultural gatherings where basic knowledge education of sickle cell disease is disclosed to community members can help dispel myths from traditions, such as the belief that sickle cell disease is a curse or result of witchcraft."

The authors Strategies for Improved Care highlight the need for an established institutional protocol for a multidisciplinary care team approach for SCD patients. It's also vital to note the authors' recognition for the need of ongoing training and education which is focused on SCD management during pregnancy.

The authors also added that increasing public knowledge could help reduce stigma and encourage more women to seek early care during their pregnancy eg. "developing a Brochure talking point for SCD in pregnancy as they currently have one for Preeclampsia and PPH would also be very helpful during educational talks in ANC and other outreaches"

The revised paper also highlighted that "having collaborative approaches that involve multiple healthcare disciplines can enhance the management of complex cases, ensuring that patients receive comprehensive care that addresses both their medical and psychosocial needs"

Finally, I'll like to say thank you once again for the invitation to review the revised manuscript. It is well suited for publication

Reviewer #3: The authors have appropriately addressed the major methodological concerns identified during the initial round of review. Specifically, purposive sampling has been detailed (lines 127-128); the saturation of data has been documented (lines 146-149); and a reflexivity plan, including other research team members interviewing subjects and triangulation through team analysis as well as recusal of the PI from subject interviewing, has been documented (lines 138-143). This journal’s qualitative methodologic standards for publication have now been met.

Before the condition is formally satisfied a final technical refinement of the bibliography will be necessary. The reference contains many parsing artifacts arisen from automatic exports of reference managers that require manual intervention to complete. There are substantial formatting failures such as:

• Raw HTML markup (*The Lancet Haematology* ) rendered visibly in Reference 3.

• The given name "Ifeanyi" left unabbreviated in Reference 8, contrary to standard Vancouver conventions.

• Academic credentials (e.g., BSN, RNM, PHN, FWACN, STTI) incorrectly parsed as author name components in Reference 14.

• Journal-section metadata ("Obstet G, Acc O") erroneously listed as author names in Reference 23.

A complete, manual citation audit for each referenced source will need to occur in order to fix the systematic export errors before the manuscript will continue to production.

Recommendation: Minor Revisions

**********

7. PLOS authors have the option to publish the peer review history of their article (what does this mean?). If published, this will include your full peer review and any attached files.

If you choose “no”, your identity will remain anonymous but your review may still be made public.

**Do you want your identity to be public for this peer review?**  For information about this choice, including consent withdrawal, please see our Privacy Policy.

Reviewer #2: **Yes:** Nonye Tochi Aghanya MSc, RN, FNP-C

Reviewer #3: **Yes:** Asrar Salem Almutairi

**********

[NOTE: If reviewer comments were submitted as an attachment file, they will be attached to this email and accessible via the submission site. Please log into your account, locate the manuscript record, and check for the action link "View Attachments". If this link does not appear, there are no attachment files.]

To ensure your figures meet our technical requirements, please review our figure guidelines: https://journals.plos.org/plosone/s/figures

You may also use PLOS’s free figure tool, NAAS, to help you prepare publication quality figures: https://journals.plos.org/plosone/s/figures#loc-tools-for-figure-preparation.

NAAS will assess whether your figures meet our technical requirements by comparing each figure against our figure specifications.

# Author comment: Author response to Decision Letter 2

**DOI:** 10.1371/journal.pone.0355018.r004

9 May 2026

Editor/Reviewer comment and Response

1. Raw HTML markup (The Lancet Hematology) rendered visibly in Reference 3: The visible HTML tags around the journal title were removed, and the reference was reformatted in standard Vancouver style. The corrected citation now begins with the article title, followed by the journal title, year, volume, issue and page range, without visible markup.

2. The given name “Ifeanyi” was left unabbreviated in Reference 8, contrary to standard Vancouver conventions: The author names in Reference 8 were manually revised to Vancouver format. “Johnson Onyekpa Ifeanyi” was corrected to initials-based formatting, and the author string was cleaned so that all given names are appropriately abbreviated.

3. Academic credentials (e.g., BSN, RNM, PHN, FWACN, STTI) were incorrectly parsed as author name components in Reference 14: Academic and professional credentials were removed from the author field in Reference 14. The duplicated/erroneously parsed author names were also cleaned, leaving the correct author list and journal citation details in Vancouver style.

4. Journal-section metadata (“Obstet G, Acc O”) was erroneously listed as author names in Reference 23: The journal-section metadata was removed from the author field in Reference 23. The reference was manually revised so that only the actual authors remain in the author position, followed by the article title and journal details.

5. A complete, manual citation audit for each referenced source is required to fix systematic export errors before the manuscript proceeds to production : Beyond the four references specifically flagged, the full reference list was manually audited for consistency with Vancouver style and production readiness. Additional corrections were made where needed, including journal-title abbreviation cleanup, page/article-number formatting, removal or standardization of inconsistent “[Internet]/Available from” export text, spacing and punctuation cleanup, and correction of minor author-name formatting issues. All such corrections were made directly in the manuscript and retained as tracked changes.

Attachment Submitted filename: Response to Reviewers.docx

# Peer review: Decision Letter 2

**DOI:** 10.1371/journal.pone.0355018.r005

**Authors:** Rehab Al-Ansari

16 Jul 2026

“Heartbreak and Frustration’’ Experiences of Healthcare Providers Caring for Pregnant Women with Sickle Cell Disease in Uganda”

PONE-D-25-50130R2

Dear Dr. Jackline Akello,

We’re pleased to inform you that your manuscript has been judged scientifically suitable for publication and will be formally accepted for publication once it meets all outstanding technical requirements.

Within one week, you’ll receive an e-mail detailing the required amendments. When these have been addressed, you’ll receive a formal acceptance letter and your manuscript will be scheduled for publication.

An invoice will be generated when your article is formally accepted. Please note, if your institution has a publishing partnership with PLOS and your article meets the relevant criteria, all or part of your publication costs will be covered. Please make sure your user information is up-to-date by logging into Editorial Manager at Editorial Manager® and clicking the ‘Update My Information' link at the top of the page. For questions related to billing, please contact billing support.

If your institution or institutions have a press office, please notify them about your upcoming paper to help maximize its impact. If they’ll be preparing press materials, please inform our press team as soon as possible -- no later than 48 hours after receiving the formal acceptance. Your manuscript will remain under strict press embargo until 2 pm Eastern Time on the date of publication. For more information, please contact onepress@plos.org.

Kind regards,

Rehab Al-Ansari

Academic Editor

PLOS One

Additional Editor Comments (optional):

Reviewers' comments:

Reviewer's Responses to Questions

**Comments to the Author**

1. If the authors have adequately addressed your comments raised in a previous round of review and you feel that this manuscript is now acceptable for publication, you may indicate that here to bypass the “Comments to the Author” section, enter your conflict of interest statement in the “Confidential to Editor” section, and submit your "Accept" recommendation.

Reviewer #2: All comments have been addressed

**********

2. Is the manuscript technically sound, and do the data support the conclusions?

The manuscript must describe a technically sound piece of scientific research with data that supports the conclusions. Experiments must have been conducted rigorously, with appropriate controls, replication, and sample sizes. The conclusions must be drawn appropriately based on the data presented.

Reviewer #2: Yes

**********

3. Has the statistical analysis been performed appropriately and rigorously?

Reviewer #2: I Don't Know

**********

4. Have the authors made all data underlying the findings in their manuscript fully available?

The PLOS Data policy requires authors to make all data underlying the findings described in their manuscript fully available without restriction, with rare exception (please refer to the Data Availability Statement in the manuscript PDF file). The data should be provided as part of the manuscript or its supporting information, or deposited to a public repository. For example, in addition to summary statistics, the data points behind means, medians and variance measures should be available. If there are restrictions on publicly sharing data—e.g. participant privacy or use of data from a third party—those must be specified.

Reviewer #2: Yes

**********

5. Is the manuscript presented in an intelligible fashion and written in standard English?

PLOS ONE does not copyedit accepted manuscripts, so the language in submitted articles must be clear, correct, and unambiguous. Any typographical or grammatical errors should be corrected at revision, so please note any specific errors here.

Reviewer #2: Yes

**********

6. Review Comments to the Author

Please use the space provided to explain your answers to the questions above. You may also include additional comments for the author, including concerns about dual publication, research ethics, or publication ethics. (Please upload your review as an attachment if it exceeds 20,000 characters)

Reviewer #2: Thank you for addressing the reviewer comments for minor revisions, which include reformatting the journal title reference, name abbreviation, and academic credentials disclosure format to comply with the required Vancouver style.

**********

7. PLOS authors have the option to publish the peer review history of their article (what does this mean?). If published, this will include your full peer review and any attached files.

If you choose “no”, your identity will remain anonymous but your review may still be made public.

**Do you want your identity to be public for this peer review?**  For information about this choice, including consent withdrawal, please see our Privacy Policy.

Reviewer #2: **Yes:** Nonye Tochi Aghanya MSc, RN, FNP-C

**********

# Editor report: Acceptance letter

**DOI:** 10.1371/journal.pone.0355018.r006

**Authors:** Rehab Al-Ansari

PONE-D-25-50130R2

PLOS One

Dear Dr. Akello,

I'm pleased to inform you that your manuscript has been deemed suitable for publication in PLOS One. Congratulations! Your manuscript is now being handed over to our production team.

At this stage, our production department will prepare your paper for publication. This includes ensuring the following:

* All references, tables, and figures are properly cited

* All relevant supporting information is included in the manuscript submission,

* There are no issues that prevent the paper from being properly typeset

You will receive further instructions from the production team, including instructions on how to review your proof when it is ready. Please keep in mind that we are working through a large volume of accepted articles, so please give us a few days to review your paper and let you know the next and final steps.

Lastly, if your institution or institutions have a press office, please let them know about your upcoming paper now to help maximize its impact. If they'll be preparing press materials, please inform our press team within the next 48 hours. Your manuscript will remain under strict press embargo until 2 pm Eastern Time on the date of publication. For more information, please contact onepress@plos.org.

You will receive an invoice from PLOS for your publication fee after your manuscript has reached the completed accept phase. If you receive an email requesting payment before acceptance or for any other service, this may be a phishing scheme. Learn how to identify phishing emails and protect your accounts at https://explore.plos.org/phishing.

If we can help with anything else, please email us at customercare@plos.org.

Thank you for submitting your work to PLOS ONE and supporting open access.

Kind regards,

PLOS ONE Editorial Office Staff

on behalf of

Dr. Rehab Al-Ansari

Academic Editor

PLOS One
